# Treatment of Desquamative Gingivitis with Free Gingival Graft: A Case Report

**DOI:** 10.5681/joddd.2010.009

**Published:** 2010-03-14

**Authors:** Mehdi Vatankhah, Mohammad Taghi Chitsazi, Masoumeh Mehdipour, Ali Taghavi Zenouz, Rasoul Estakhri

**Affiliations:** ^1^ Instructor, Department of Oral Medicine, Faculty of Dentistry, Tabriz University of Medical Sciences, Tabriz, Iran; ^2^ Associate Professor, Department of Periodontics, Faculty of Dentistry, Tabriz University of Medical Sciences, Tabriz, Iran; ^3^ Assistant Professor, Department of Oral Medicine, Faculty of Dentistry, Tabriz University of Medical Sciences, Tabriz, Iran; ^4^ Associate Professor, Department of Pathology, Imam Reza Hospital, Tabriz University of Medical Sciences, Tabriz, Iran

**Keywords:** Desquamative gingivitis, free gingival graft, lichen planus

## Abstract

Recalcitrant gingival erythematous lichen planus lesions comprise a considerable therapeutic problem. This case of chronic desquamative gingivitis in a 25-year-old woman with erosive oral lichen planus was treated with topical and systemic corti-costeroid administration, followed by placement of a free gingival graft on right upper quadrant. Although recurrence of the lesions was observed following both treatment modalities, free gingival graft despite being an aggressive therapy, proved more effective and with fewer side effects compared with topical or systemic steroid therapy, and seems to be a promising treatment modality with the benefit of more stable results, among others.

## Introduction

Desquamative gingivitis (DG) is the clinical term given to the gingival manifestation of mucocutaneous diseases. It is characterized by an erythematous, glazed, friable and hemorrhagic gingiva, which can be accompanied by pain.^1^ Desquamative gingivitis is believed to be a clinical sign of certain mucocutaneous diseases rather than a distinct pathologic entity.^2^ An accurate diagnosis of the underlying disease of chronic desquamative gingivitis can be made on the basis of careful history and clinical observation, light microscopic examination of gingival biopsy specimens, and immunopathologic and follow-up findings. The significance of early diagnosis in the therapeutical management of the patients is emphasized. The response to topical corticosteroids as well as systemic corticosteroids and dapsone or sulfapyridine has been gratifying. The identification of the underlying disease in chronic desquamative gingivitis is important and the contribution of the dentist in early diagnosis and prompt therapeutical care is of great value.^3^

Despite the availability of many therapeutic agents that claim to reduce severity, no intervention that is completely successful for treatment of desquamative gingivitis exists. The gingival lesions are usually treated by improved oral hygiene measures and occlusive topical and systemic corticosteroid therapy.

This paper presents a case of chronic desquamative gingivitis in a young female patient with erosive oral lichen planus and focuses on the importance of connective tissue grafting from palate for treatment.

## Case Report

A 25-year-old female patient with a chief complaint of oral discomfort and soreness of the gingiva was referred to the Department of Oral Medicine, Tabriz University of Medical Sciences, for treatment.

Oral examination showed the presence of multiple bilateral erythematous and desquamative areas on both upper and lower gingival mucosa. The most symptomatic gingival regions in the patient were the labial aspect of the anterior and the buccal aspects of the posterior maxilla, and the buccal mandibular premolar areas (Figure 1a). Because of the pain, home dental hygiene was difficult and discouraging for the patient.


Figure 1. Desquamative gingivitis at first day (a); gingival flap (b) and removal of involved tissue (c); the site (d) of palatal graft (e); fixation of graft by sutures (f).

**a:**
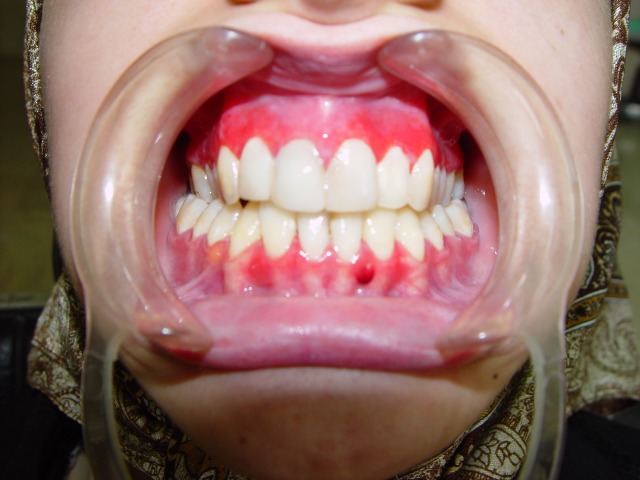

**b:**
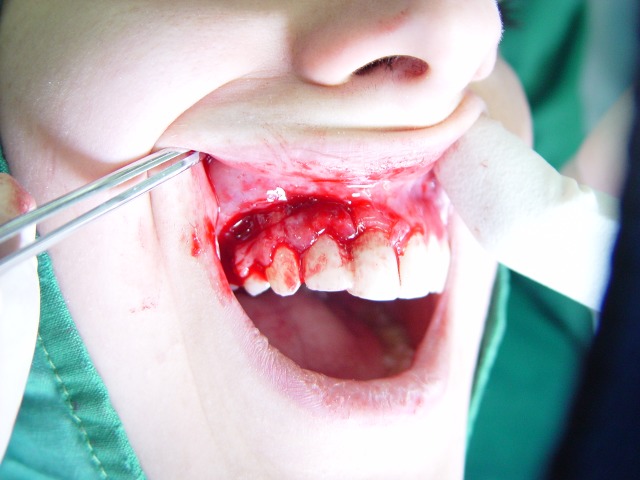

**c:**
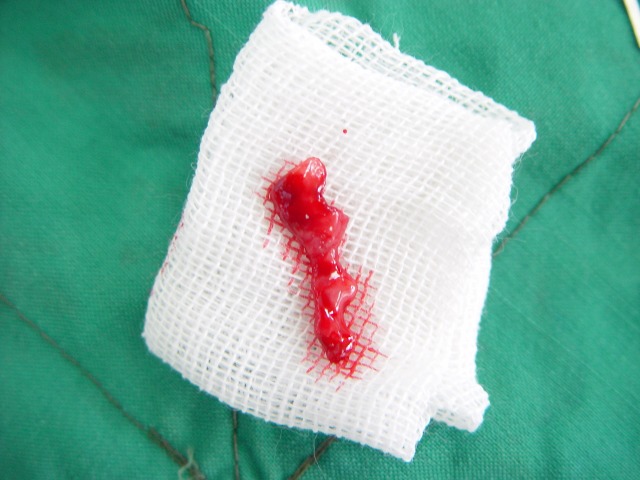

**d:**
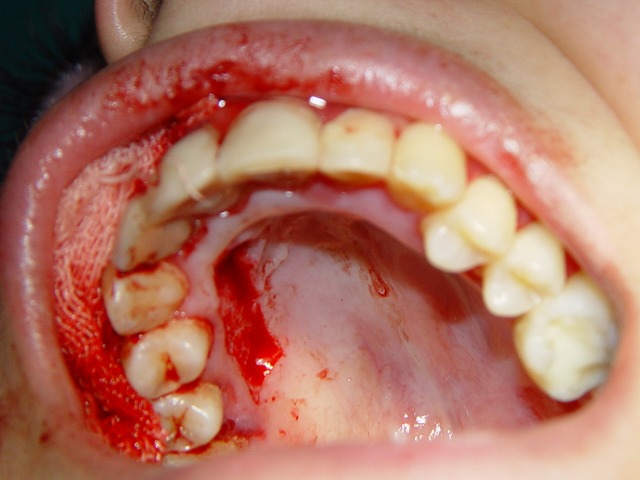

**e:**
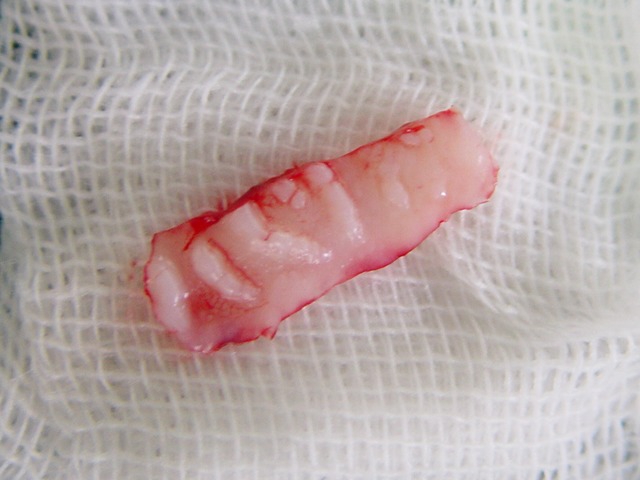

**f:**
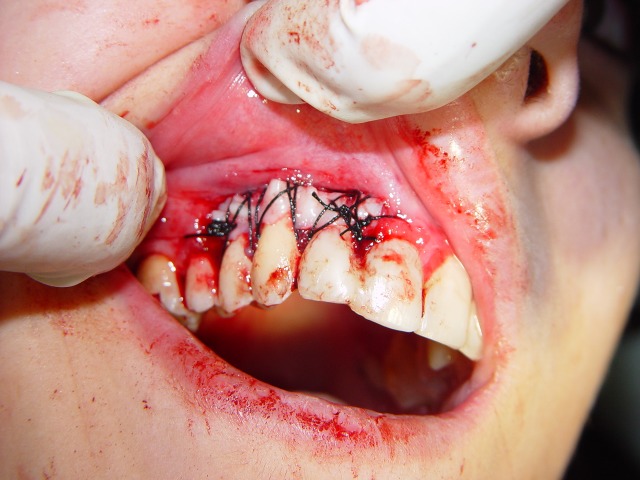

Considering the clinical findings and the history, erosive lichen planus was suspected. Identification of the main disease was based on the following criteria: Detailed clinical examination of the buccal and extrabuccal lesions, biopsy for pathoanatomical examination, and examination with direct immunofluorescence. The histopathological examination of the gingival lesion showed a flattened epithelium with liquefaction of the basal layer and juxtaepithelial areas of chronic inflammatory infiltrate. Direct immunofluorescence showed heavy deposits of fibrin at the dermo-epidermal junction. Deposits of IgG and C3 were found in the colloid bodies. According to both clinical and histopathological patterns observed, the diagnosis was gingival erosive lichen planus.

Moderate potent topical steroid (triamcinolon b.d) for two weeks was started. Within two weeks, the oral lesions improved markedly and treatment was discontinuedafter three weeks. The patient also received nystatin orally for prevention of candidiasis. The lesions recurred afterthe medication was discontinued. According to the guidelines for systemic corticosteroid treatment,^4^ 50 mg systemic methylprednisolone (1 mg/kg per day) was given for six weeks with partial response only.

At this point, another treatment modality, free gingival graft surgery, was indicated. After discussing the benefits and complications, the patient was treated by placement of a free gingival graft on the right upper quadrant. Following intraoral disinfection with 0.12 chlorhexidine mouthrinse, local anesthesia (Xylocaine) was administered. The first surgical phase involved preparation of the recipient bed apical to desquamative gingivitis area. A horizontal full thickness incision was made with scalpel 15 along the mucogingival junction extended one tooth medially and distally from the affected area (Figure 1b,c). The gingival margin of the vestibular mucosa was fixed to the periosteum using silk sutures. Free gingival graft was harvested from the palate and was adapted into recipient bed. The free gingival graft was secured using atraumatic sutures and sharply curved needle (Figure 1d,e,f). The dressing and the sutures were removed day 10-14. For the left side, Adcortyl (triamcinolon in Orabase b.d.) was started for 4 weeks, which was unsuccessful. The patient was followed up every week, for the first one month. Other evaluations were performed every 3 months until 9 months. One month after surgery, the color and the appearance of the area with the free gingival graft was similar to the adjacent gingiva, giving a good esthetic result (Figure 2a). In the maxillary left quadrant (conventional therapy with Adcortyl), gingiva exhibited recurrence of the initial redness and desquamation (but not severe).


Figure 2. Comparison of surgical side (maxillary right quadrant) with medication therapy side (maxillary left quadrant) (a); Recurrence of desquamation (b); Oral candidiasis (thrush) following Fluocinonide therapy (c).

**a:**
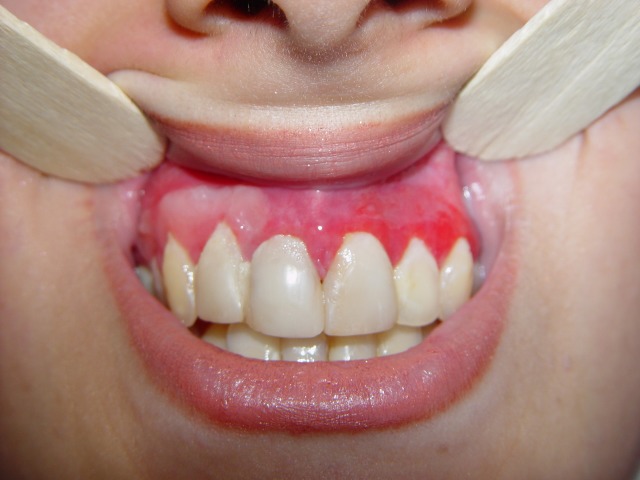

**b:**
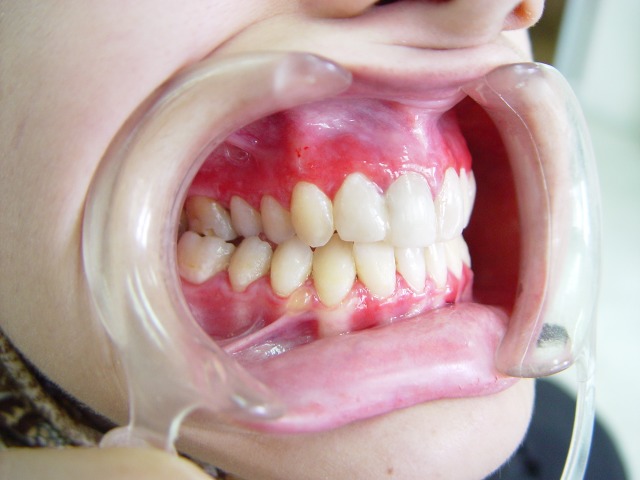

**c:**
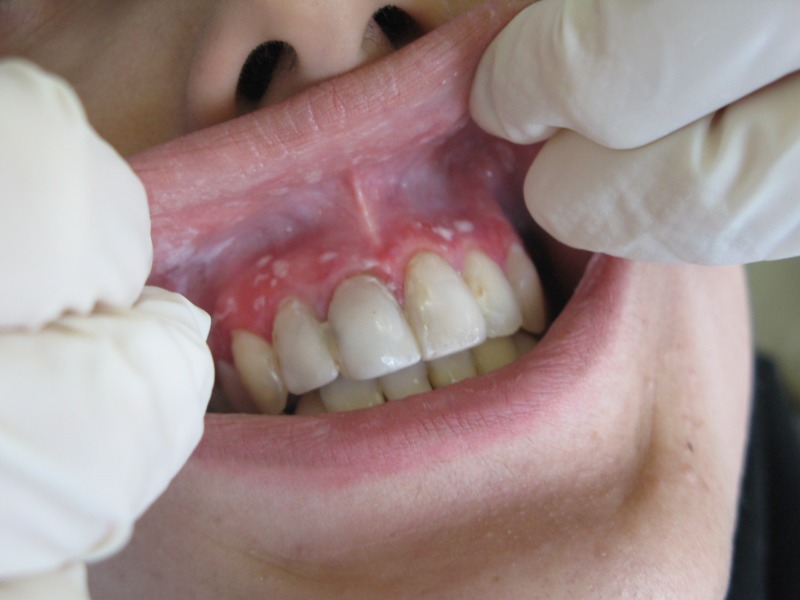

The results were stable 9 months after the initial surgery. After this period, however, the patient complained of bleeding, pain, and increase in the severity of peeling of the gums on right and left maxillary quadrants (Figure 2b). At this time, occlusive topical steroid therapy and antifungal therapy was performed (Figure 2c). A custom tray was produced for the patient for the application of 0.025% Flucinonide plus Clotrimazol ointment. The mixture was easily produced using a blender. A good seal was established between the tray and the gingival tissue. The paste was applied with the tray for 20 minutes 3 times daily (after breakfast, after lunch, and after the evening meal) for ten days; and was tapered within 3 weeks.

The patient became completely asymptomatic after the topical treatment regarding DG. However, the patient had gained weight by 3 kg and started to suffer from candidiasis despite using Clotrimazol (Figure 2c). Nystatin suspension was administered for 14 days, during which thrush disappeared. The patient was asymptomatic for 2 months. However, desquamative gingivitis gradually returned.

## Discussion

In the present case, in addition to the free gingival graft, topical application of 0.025% Flucinonide was introduced as an adjunct modality. In this case, treatment with free gingival graft was more effective, had fewer side effects and was more stable than topical or systemic steroid therapy.

In treatment of lichen planus, surgical management including cryosurgery and carbon dioxide laser has been suggested. Despite this, surgical excision is not recommended as the first choice of treatment due to the inflammatory condition which may recur.^5^ The use of topical corticosteroids with other types of desquamative gingival conditions such as erosive lichen planus or cicatricial pemphigoid is common.^6-8^

Although the patient did not show improvement of gingival lesions while using topical and systemic steroid, the shorter treatment period does reduce the risk of side effects. A risk/benefit analysis should be undertaken when considering an increase in the dosage of any medication.

In patients with lichen planus, free gingival grafts have been used successfully to reinforce marginal dental soft tissues and to help stabilize recession.^9,10^

et al^11^ used an autologous grafting material for regenerating gingival tissue in the maxillary left and mandibular right quadrants of a patient with chronic desquamative gingivitis. Six months post-surgery in both treated areas, there were gains in keratinized gingiva and no signs of gingival inflammation compared to presurgery. Katz et al^12^ suggest that trauma to the oral mucosa by a surgical procedure may induce the formation of new lesions at these sites.

In desquamative gingivitis, there is a disturbance in the ground substance of the gingival connective tissue. The possible influence of the female sex hormones in inducing the changes of the ground substance is considered.^13^

Precipitating factors that resulted in an exacerbation of the disease were frequently noted in this case and included stress, foods, dental procedures, and poor oral hygiene.

Treatment of oral lichen planus patients with desquamative gingivitis by use of free gingival graft is an aggressive therapy but more effective, with fewer side effects and more stable (as the most important benefit) compared to topical or systemic steroids. In the present case, despite full thickness removal of gingiva, recurrence occurred, with lesions similar in shape and severity to the primary lesions. However, the recurrence appeared after a longer period compared to the conventional therapy. The probable etiology and pathogenesis of this “specific recurrence” should therefore be assessed further.
